# *N*′-Cyclo­dodecyl­idene­pyridine-4-carbohydrazide

**DOI:** 10.1107/S1600536812011774

**Published:** 2012-03-28

**Authors:** Andreas Lemmerer, Joel Bernstein, Volker Kahlenberg

**Affiliations:** a Molecular Sciences Institute, School of Chemistry, University of the Witwatersrand, Johannesburg, PO Wits 2050, South Africa; b Faculty of Science, NYU Abu Dhabi, PO Box 129188, Abu Dhabi, United Arab Emirates; c Institute of Mineralogy and Petrography, University of Innsbruck, Innsbruck 6020, Austria

## Abstract

The title compound, C_18_H_27_N_3_O, is a derivative of the anti­tuberculosis drug isoniazid (systematic name: pyridine-4-carbohydrazidei). The crystal structure consists of repeating *C*(4) chains along the *b* axis, formed by N—H⋯O hydrogen bonds with adjacent amide functional groups that are related by a *b*-glide plane. The cyclo­dodecyl ring has the same approximately ‘square’ conformation, as seen in the parent hydro­carbon cyclo­dodecane.

## Related literature

For hydrogen-bonding motifs, see: Bernstein *et al.* (1995 ▶). For cyclo­alkane ring conformations, see: Dale (1966 ▶).
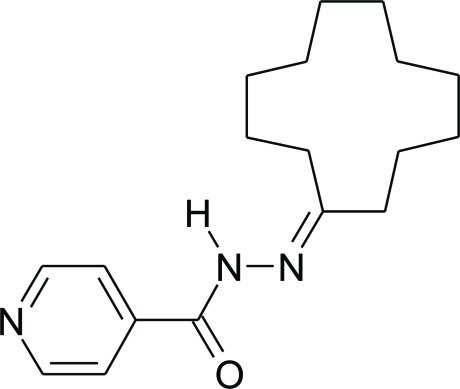


## Experimental

### 

#### Crystal data

C_18_H_27_N_3_O
*M* *_r_* = 301.43
Orthorhombic, 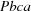

*a* = 14.8450 (6) Å
*b* = 8.0980 (4) Å
*c* = 27.3910 (11) Å
*V* = 3292.8 (2) Å^3^
*Z* = 8
Cu *K*α radiation
μ = 0.60 mm^−1^
*T* = 100 K
0.44 × 0.34 × 0.2 mm

#### Data collection

Oxford Diffraction Xcalibur Ruby Gemini ultra diffractometer
Absorption correction: multi-scan (*CrysAlis PRO*; Oxford Diffraction, 2006 ▶) *T*_min_ = 0.779, *T*_max_ = 0.890
23848 measured reflections
2931 independent reflections
2535 reflections with *I* > 2σ(*I*)
*R*_int_ = 0.048

#### Refinement

*R*[*F* ^2^ > 2σ(*F* ^2^)] = 0.040
*wR*(*F* ^2^) = 0.111
*S* = 1.08
2931 reflections
203 parameters
H atoms treated by a mixture of independent and constrained refinement
Δρ_max_ = 0.17 e Å^−3^
Δρ_min_ = −0.20 e Å^−3^

### 

Data collection: *CrysAlis PRO* (Oxford Diffraction, 2006 ▶); cell refinement: *CrysAlis PRO*; data reduction: *CrysAlis PRO*; program(s) used to solve structure: *SHELXS97* (Sheldrick, 2008 ▶); program(s) used to refine structure: *SHELXL97* (Sheldrick, 2008 ▶); molecular graphics: *ORTEP-3 for Windows* (Farrugia, 1997 ▶) and *DIAMOND* (Brandenburg, 1999 ▶); software used to prepare material for publication: *WinGX* (Farrugia, 1999 ▶) and *PLATON* (Spek, 2009 ▶).

## Supplementary Material

Crystal structure: contains datablock(s) global, I. DOI: 10.1107/S1600536812011774/fj2532sup1.cif

Supplementary material file. DOI: 10.1107/S1600536812011774/fj2532Isup2.mol

Structure factors: contains datablock(s) I. DOI: 10.1107/S1600536812011774/fj2532Isup3.hkl

Supplementary material file. DOI: 10.1107/S1600536812011774/fj2532Isup4.cml

Additional supplementary materials:  crystallographic information; 3D view; checkCIF report

## Figures and Tables

**Table 1:** Hydrogen-bond geometry (Å, °)

*D*—H⋯*A*	*D*—H	H⋯*A*	*D*⋯*A*	*D*—H⋯*A*
N1—H1⋯O1^i^	0.88 (2)	2.15 (2)	3.0122 (15)	164.7 (16)

Symmetry code: (i) 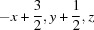.

**Fig. 1.**
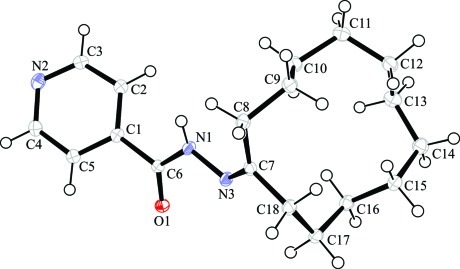
The asymmetric unit of (I) showing the atomic numbering scheme. Displacement ellipsoids are shown at the 50% probability level.

**Fig. 2.**
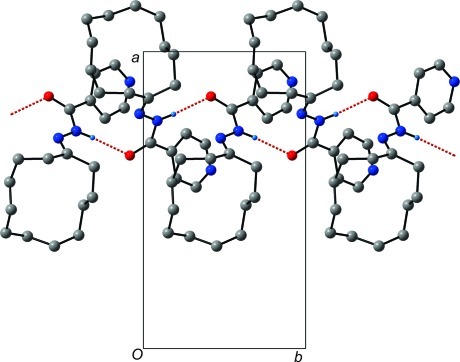
Hydrogen bonding chain showing the C(4) hydrogen bonded chains. Intermolecular N—H···O hydrogen bonds are shown as dashed red lines.

## supplementary crystallographic information

### Comment

Fig. 1 shows the atomic numbering scheme of the title compound. The amide functional groups form a torsion angle of 38.54 (17)° with the pyridine ring. Fig. 2 shows the *C*(4) (Bernstein *et al.*, 1995) hydrogen bonded ring formed with adjacent amide functional groups, leading to a chain along the *b*-axis. The cyclododecyl ring has a square conformation, as seen in the related cycloalkane C_12_H_24_ ring (Dale, 1966).

### Experimental

A stoichiometric amount in the ratio of 1:1 of isonicotinic acid hydrazide to cyclododecanone was dissolved in 5 ml of methanol. The solution was refluxed for a few hours, and left to cool to room temperature. Colourless, block-like crystals were harvested after slow evaporation over a few days at ambient conditions.

### Refinement

The C-bound H atoms were geometrically placed (C—H bond lengths of 0.95 (aromatic CH) and 0.99 (methylene CH_2_) Å) and refined as riding with *U*_iso_(H) = 1.2*U*_eq_(C). The N-bound H atoms were located in the difference map and coordinates refined freely together with their isotropic thermal parameters.

### Crystal data

**Table:**

C_18_H_27_N_3_O	*F*(000) = 1312
*M**_r_* = 301.43	*D*_x_ = 1.216 Mg m^−^^3^
Orthorhombic, *P**b**c**a*	Cu *K*α radiation, λ = 1.5418 Å
Hall symbol: -P 2ac 2ab	Cell parameters from 9464 reflections
*a* = 14.8450 (6) Å	θ = 3.0–67.5°
*b* = 8.0980 (4) Å	µ = 0.60 mm^−^^1^
*c* = 27.3910 (11) Å	*T* = 100 K
*V* = 3292.8 (2) Å^3^	Block, colourless
*Z* = 8	0.44 × 0.34 × 0.2 mm

### Data collection

**Table:**

Oxford Diffraction Xcalibur Ruby Gemini ultra diffractometer	2535 reflections with *I* > 2σ(*I*)
ω scans	*R*_int_ = 0.048
Absorption correction: multi-scan (*CrysAlis PRO*; Oxford Diffraction, 2006)	θ_max_ = 67.0°, θ_min_ = 3.2°
*T*_min_ = 0.779, *T*_max_ = 0.890	*h* = −17→16
23848 measured reflections	*k* = −9→9
2931 independent reflections	*l* = −32→32

### Refinement

**Table:**

Refinement on *F*^2^	0 restraints
Least-squares matrix: full	H atoms treated by a mixture of independent and constrained refinement
*R*[*F*^2^ > 2σ(*F*^2^)] = 0.040	*w* = 1/[σ^2^(*F*_o_^2^) + (0.0722*P*)^2^ + 0.6785*P*] where *P* = (*F*_o_^2^ + 2*F*_c_^2^)/3
*wR*(*F*^2^) = 0.111	(Δ/σ)_max_ = 0.001
*S* = 1.08	Δρ_max_ = 0.17 e Å^−^^3^
2931 reflections	Δρ_min_ = −0.20 e Å^−^^3^
203 parameters	

### Special details

**Table:**

Experimental. Empirical absorption correction using spherical harmonics, implemented in SCALE3 ABSPACK scaling algorithm in CrysAlisPro.
Geometry. All e.s.d.'s (except the e.s.d. in the dihedral angle between two l.s. planes) are estimated using the full covariance matrix. The cell e.s.d.'s are taken into account individually in the estimation of e.s.d.'s in distances, angles and torsion angles; correlations between e.s.d.'s in cell parameters are only used when they are defined by crystal symmetry. An approximate (isotropic) treatment of cell e.s.d.'s is used for estimating e.s.d.'s involving l.s. planes.

### Fractional atomic coordinates and isotropic or equivalent isotropic displacement parameters (Å^2^)

**Table:**

	*x*	*y*	*z*	*U*_iso_*/*U*_eq_	
C1	0.83538 (9)	0.67344 (15)	0.71252 (5)	0.0140 (3)	
C2	0.77610 (9)	0.76355 (16)	0.74142 (5)	0.0151 (3)	
H2	0.713	0.7447	0.7397	0.018*	
C3	0.81105 (9)	0.88164 (16)	0.77286 (5)	0.0171 (3)	
H3	0.7701	0.9417	0.7927	0.02*	
C4	0.95549 (9)	0.82425 (17)	0.74991 (5)	0.0185 (3)	
H4	1.0183	0.8447	0.7527	0.022*	
C5	0.92753 (9)	0.70136 (16)	0.71813 (5)	0.0157 (3)	
H5	0.9702	0.6374	0.7005	0.019*	
C6	0.80631 (9)	0.54546 (16)	0.67606 (4)	0.0131 (3)	
C7	0.65632 (8)	0.52936 (16)	0.57794 (5)	0.0144 (3)	
C8	0.61244 (9)	0.69684 (16)	0.57370 (5)	0.0160 (3)	
H8A	0.6251	0.7436	0.541	0.019*	
H8B	0.6386	0.7721	0.5984	0.019*	
C9	0.50972 (9)	0.68614 (17)	0.58126 (5)	0.0179 (3)	
H9A	0.4828	0.7957	0.5746	0.022*	
H9B	0.4843	0.607	0.5574	0.022*	
C10	0.48338 (9)	0.63169 (18)	0.63275 (5)	0.0208 (3)	
H10A	0.5026	0.7177	0.6562	0.025*	
H10B	0.5163	0.5289	0.6408	0.025*	
C11	0.38197 (9)	0.60107 (18)	0.63905 (5)	0.0221 (3)	
H11A	0.3691	0.5856	0.6742	0.027*	
H11B	0.349	0.7008	0.6281	0.027*	
C12	0.34546 (9)	0.45158 (17)	0.61105 (5)	0.0202 (3)	
H12A	0.2788	0.4539	0.6123	0.024*	
H12B	0.3636	0.4613	0.5764	0.024*	
C13	0.37813 (10)	0.28492 (17)	0.63069 (5)	0.0219 (3)	
H13A	0.4364	0.3016	0.6475	0.026*	
H13B	0.3343	0.2447	0.6552	0.026*	
C14	0.39013 (10)	0.15214 (17)	0.59157 (6)	0.0224 (3)	
H14A	0.3336	0.1432	0.5726	0.027*	
H14B	0.4004	0.0446	0.6078	0.027*	
C15	0.46814 (9)	0.18511 (17)	0.55621 (5)	0.0190 (3)	
H15A	0.4653	0.1034	0.5294	0.023*	
H15B	0.4601	0.2961	0.5417	0.023*	
C16	0.56119 (9)	0.17631 (16)	0.57975 (5)	0.0178 (3)	
H16A	0.5722	0.0615	0.5906	0.021*	
H16B	0.5615	0.2476	0.6091	0.021*	
C17	0.63832 (9)	0.22928 (17)	0.54617 (5)	0.0178 (3)	
H17A	0.6962	0.1979	0.5615	0.021*	
H17B	0.6333	0.1683	0.515	0.021*	
C18	0.63976 (9)	0.41420 (17)	0.53531 (5)	0.0168 (3)	
H18A	0.687	0.4349	0.5105	0.02*	
H18B	0.5813	0.4443	0.5204	0.02*	
N1	0.73263 (7)	0.58502 (14)	0.64919 (4)	0.0148 (3)	
H1	0.7106 (12)	0.686 (2)	0.6498 (6)	0.024 (4)*	
N2	0.89901 (8)	0.91633 (14)	0.77697 (4)	0.0191 (3)	
N3	0.70898 (7)	0.47642 (14)	0.61174 (4)	0.0148 (3)	
O1	0.84951 (6)	0.41677 (11)	0.67137 (3)	0.0159 (2)	

### Atomic displacement parameters (Å^2^)

**Table:**

	*U*^11^	*U*^22^	*U*^33^	*U*^12^	*U*^13^	*U*^23^
C1	0.0179 (6)	0.0130 (6)	0.0113 (6)	0.0000 (5)	−0.0025 (5)	0.0028 (5)
C2	0.0164 (6)	0.0153 (6)	0.0136 (6)	0.0009 (5)	−0.0012 (5)	0.0028 (5)
C3	0.0202 (7)	0.0163 (6)	0.0147 (6)	0.0022 (5)	−0.0002 (5)	0.0004 (5)
C4	0.0180 (6)	0.0203 (7)	0.0171 (6)	−0.0029 (5)	−0.0012 (5)	0.0013 (5)
C5	0.0174 (7)	0.0160 (6)	0.0136 (6)	−0.0001 (5)	0.0007 (5)	0.0018 (5)
C6	0.0146 (6)	0.0135 (6)	0.0113 (6)	−0.0011 (5)	0.0014 (5)	0.0015 (5)
C7	0.0128 (6)	0.0164 (7)	0.0141 (6)	−0.0024 (5)	0.0012 (5)	−0.0004 (5)
C8	0.0169 (7)	0.0164 (6)	0.0146 (6)	−0.0010 (5)	−0.0031 (5)	0.0005 (5)
C9	0.0166 (7)	0.0182 (7)	0.0190 (7)	0.0013 (5)	−0.0023 (5)	−0.0009 (5)
C10	0.0189 (7)	0.0257 (7)	0.0178 (7)	−0.0006 (6)	0.0011 (5)	−0.0046 (6)
C11	0.0189 (7)	0.0247 (7)	0.0227 (7)	0.0018 (6)	0.0041 (6)	−0.0035 (6)
C12	0.0160 (6)	0.0228 (7)	0.0220 (7)	0.0016 (5)	0.0001 (5)	−0.0004 (6)
C13	0.0215 (7)	0.0240 (7)	0.0201 (7)	0.0010 (6)	0.0031 (6)	0.0033 (6)
C14	0.0208 (7)	0.0186 (7)	0.0278 (8)	−0.0017 (5)	0.0007 (6)	0.0002 (6)
C15	0.0200 (7)	0.0179 (7)	0.0192 (7)	−0.0008 (5)	−0.0015 (5)	−0.0034 (5)
C16	0.0203 (7)	0.0141 (6)	0.0192 (7)	0.0007 (5)	−0.0027 (5)	0.0002 (5)
C17	0.0174 (6)	0.0182 (7)	0.0177 (7)	0.0016 (5)	−0.0028 (5)	−0.0048 (5)
C18	0.0172 (6)	0.0197 (7)	0.0134 (6)	−0.0012 (5)	−0.0008 (5)	−0.0018 (5)
N1	0.0168 (5)	0.0131 (6)	0.0145 (5)	0.0014 (4)	−0.0039 (4)	−0.0029 (4)
N2	0.0234 (6)	0.0178 (6)	0.0161 (6)	−0.0027 (5)	−0.0025 (5)	−0.0003 (4)
N3	0.0159 (5)	0.0157 (5)	0.0127 (5)	−0.0020 (4)	−0.0007 (4)	−0.0027 (4)
O1	0.0165 (5)	0.0145 (5)	0.0166 (5)	0.0011 (3)	0.0001 (4)	−0.0001 (4)

### Geometric parameters (Å, º)

**Table:**

C1—C2	1.3905 (19)	C11—C12	1.532 (2)
C1—C5	1.3950 (18)	C11—H11A	0.99
C1—C6	1.5026 (17)	C11—H11B	0.99
C2—C3	1.3875 (19)	C12—C13	1.5317 (19)
C2—H2	0.95	C12—H12A	0.99
C3—N2	1.3404 (18)	C12—H12B	0.99
C3—H3	0.95	C13—C14	1.529 (2)
C4—N2	1.3447 (19)	C13—H13A	0.99
C4—C5	1.3857 (19)	C13—H13B	0.99
C4—H4	0.95	C14—C15	1.533 (2)
C5—H5	0.95	C14—H14A	0.99
C6—O1	1.2304 (16)	C14—H14B	0.99
C6—N1	1.3567 (17)	C15—C16	1.5262 (18)
C7—N3	1.2853 (17)	C15—H15A	0.99
C7—C8	1.5091 (18)	C15—H15B	0.99
C7—C18	1.5144 (18)	C16—C17	1.5301 (19)
C8—C9	1.5414 (18)	C16—H16A	0.99
C8—H8A	0.99	C16—H16B	0.99
C8—H8B	0.99	C17—C18	1.5268 (19)
C9—C10	1.5286 (19)	C17—H17A	0.99
C9—H9A	0.99	C17—H17B	0.99
C9—H9B	0.99	C18—H18A	0.99
C10—C11	1.5354 (19)	C18—H18B	0.99
C10—H10A	0.99	N1—N3	1.3961 (15)
C10—H10B	0.99	N1—H1	0.88 (2)
			
C2—C1—C5	118.21 (12)	C13—C12—H12A	108.7
C2—C1—C6	123.96 (12)	C11—C12—H12A	108.7
C5—C1—C6	117.82 (12)	C13—C12—H12B	108.7
C3—C2—C1	118.59 (12)	C11—C12—H12B	108.7
C3—C2—H2	120.7	H12A—C12—H12B	107.6
C1—C2—H2	120.7	C14—C13—C12	114.23 (12)
N2—C3—C2	124.11 (12)	C14—C13—H13A	108.7
N2—C3—H3	117.9	C12—C13—H13A	108.7
C2—C3—H3	117.9	C14—C13—H13B	108.7
N2—C4—C5	123.89 (13)	C12—C13—H13B	108.7
N2—C4—H4	118.1	H13A—C13—H13B	107.6
C5—C4—H4	118.1	C13—C14—C15	114.11 (11)
C4—C5—C1	118.65 (12)	C13—C14—H14A	108.7
C4—C5—H5	120.7	C15—C14—H14A	108.7
C1—C5—H5	120.7	C13—C14—H14B	108.7
O1—C6—N1	124.31 (12)	C15—C14—H14B	108.7
O1—C6—C1	120.26 (11)	H14A—C14—H14B	107.6
N1—C6—C1	115.42 (11)	C16—C15—C14	114.11 (12)
N3—C7—C8	128.15 (12)	C16—C15—H15A	108.7
N3—C7—C18	116.66 (12)	C14—C15—H15A	108.7
C8—C7—C18	115.09 (11)	C16—C15—H15B	108.7
C7—C8—C9	111.48 (11)	C14—C15—H15B	108.7
C7—C8—H8A	109.3	H15A—C15—H15B	107.6
C9—C8—H8A	109.3	C15—C16—C17	114.22 (11)
C7—C8—H8B	109.3	C15—C16—H16A	108.7
C9—C8—H8B	109.3	C17—C16—H16A	108.7
H8A—C8—H8B	108	C15—C16—H16B	108.7
C10—C9—C8	113.16 (11)	C17—C16—H16B	108.7
C10—C9—H9A	108.9	H16A—C16—H16B	107.6
C8—C9—H9A	108.9	C18—C17—C16	113.74 (11)
C10—C9—H9B	108.9	C18—C17—H17A	108.8
C8—C9—H9B	108.9	C16—C17—H17A	108.8
H9A—C9—H9B	107.8	C18—C17—H17B	108.8
C9—C10—C11	113.64 (12)	C16—C17—H17B	108.8
C9—C10—H10A	108.8	H17A—C17—H17B	107.7
C11—C10—H10A	108.8	C7—C18—C17	117.13 (11)
C9—C10—H10B	108.8	C7—C18—H18A	108
C11—C10—H10B	108.8	C17—C18—H18A	108
H10A—C10—H10B	107.7	C7—C18—H18B	108
C12—C11—C10	114.72 (11)	C17—C18—H18B	108
C12—C11—H11A	108.6	H18A—C18—H18B	107.3
C10—C11—H11A	108.6	C6—N1—N3	116.91 (11)
C12—C11—H11B	108.6	C6—N1—H1	120.5 (12)
C10—C11—H11B	108.6	N3—N1—H1	120.4 (12)
H11A—C11—H11B	107.6	C3—N2—C4	116.42 (12)
C13—C12—C11	114.10 (12)	C7—N3—N1	118.17 (11)
			
C5—C1—C2—C3	2.74 (18)	C11—C12—C13—C14	−146.98 (12)
C6—C1—C2—C3	−178.29 (12)	C12—C13—C14—C15	68.58 (16)
C1—C2—C3—N2	0.7 (2)	C13—C14—C15—C16	67.05 (16)
N2—C4—C5—C1	1.9 (2)	C14—C15—C16—C17	−173.04 (11)
C2—C1—C5—C4	−3.94 (18)	C15—C16—C17—C18	70.36 (15)
C6—C1—C5—C4	177.03 (11)	N3—C7—C18—C17	33.46 (17)
C2—C1—C6—O1	−140.43 (13)	C8—C7—C18—C17	−149.87 (11)
C5—C1—C6—O1	38.54 (17)	C16—C17—C18—C7	64.33 (15)
C2—C1—C6—N1	41.05 (17)	O1—C6—N1—N3	−4.55 (19)
C5—C1—C6—N1	−139.98 (12)	C1—C6—N1—N3	173.91 (10)
N3—C7—C8—C9	−110.81 (15)	C2—C3—N2—C4	−2.77 (19)
C18—C7—C8—C9	72.97 (14)	C5—C4—N2—C3	1.44 (19)
C7—C8—C9—C10	64.83 (15)	C8—C7—N3—N1	−2.22 (19)
C8—C9—C10—C11	−173.28 (11)	C18—C7—N3—N1	173.95 (11)
C9—C10—C11—C12	68.04 (16)	C6—N1—N3—C7	−161.67 (12)
C10—C11—C12—C13	68.83 (16)		

### Hydrogen-bond geometry (Å, º)

**Table:**

*D*—H···*A*	*D*—H	H···*A*	*D*···*A*	*D*—H···*A*
N1—H1···O1^i^	0.88 (2)	2.15 (2)	3.0122 (15)	164.7 (16)

Symmetry code: (i) −*x*+3/2, *y*+1/2, *z*.
